# Supplementary material for: Prognostic significance of frequent CLDN18-ARHGAP26/6 fusion in gastric signet-ring cell cancer
Source: Nat Commun. 2018 Jun 30;9:2447. doi: 10.1038/s41467-018-04907-0 (PMC6026495; doi:10.1038/s41467-018-04907-0)
Supplement: Supplementary file 1 — Supplementary Information [file 41467_2018_4907_MOESM1_ESM.pdf]

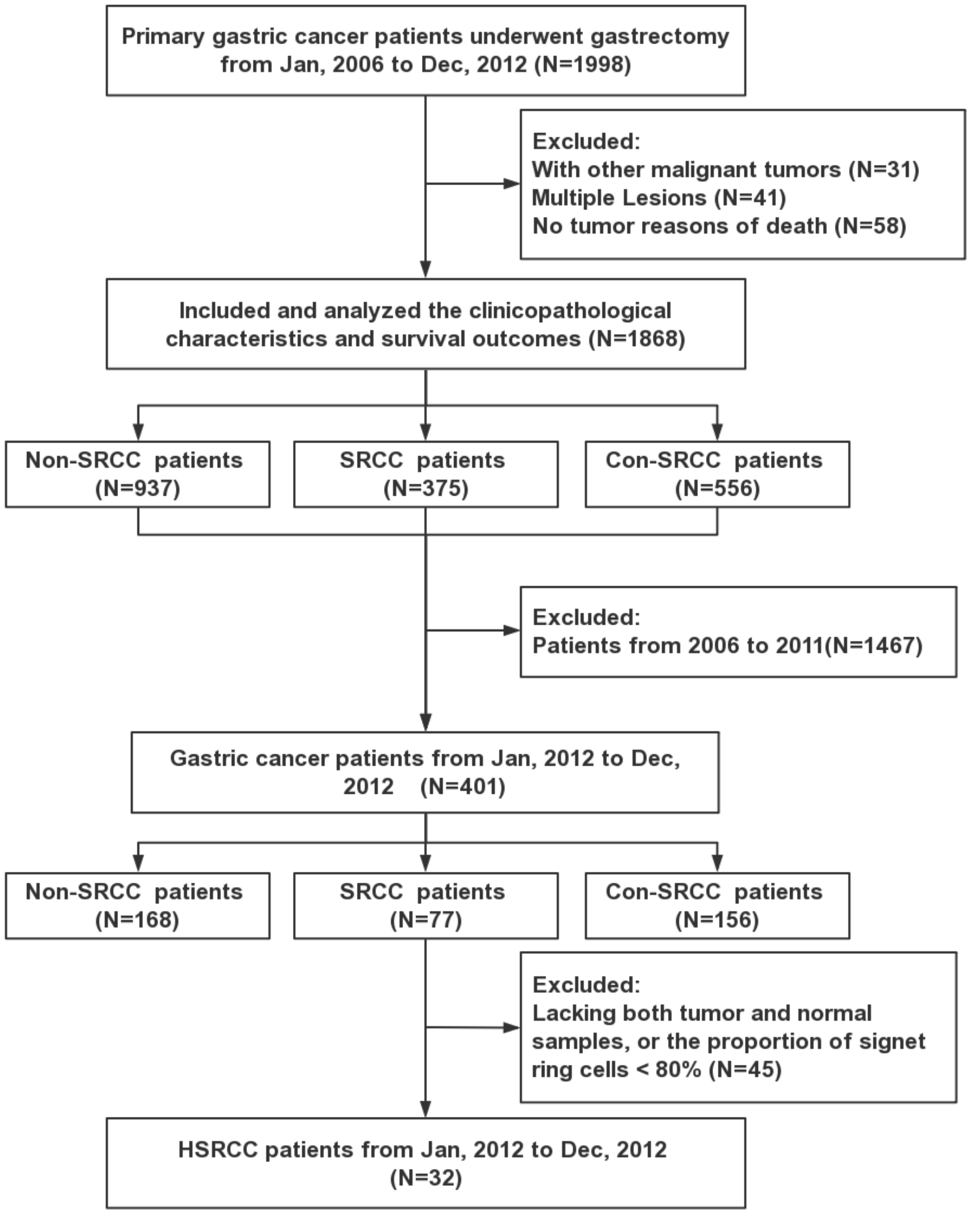

Supplementary Figure 1: Flowchart of patients' inclusion/exclusion

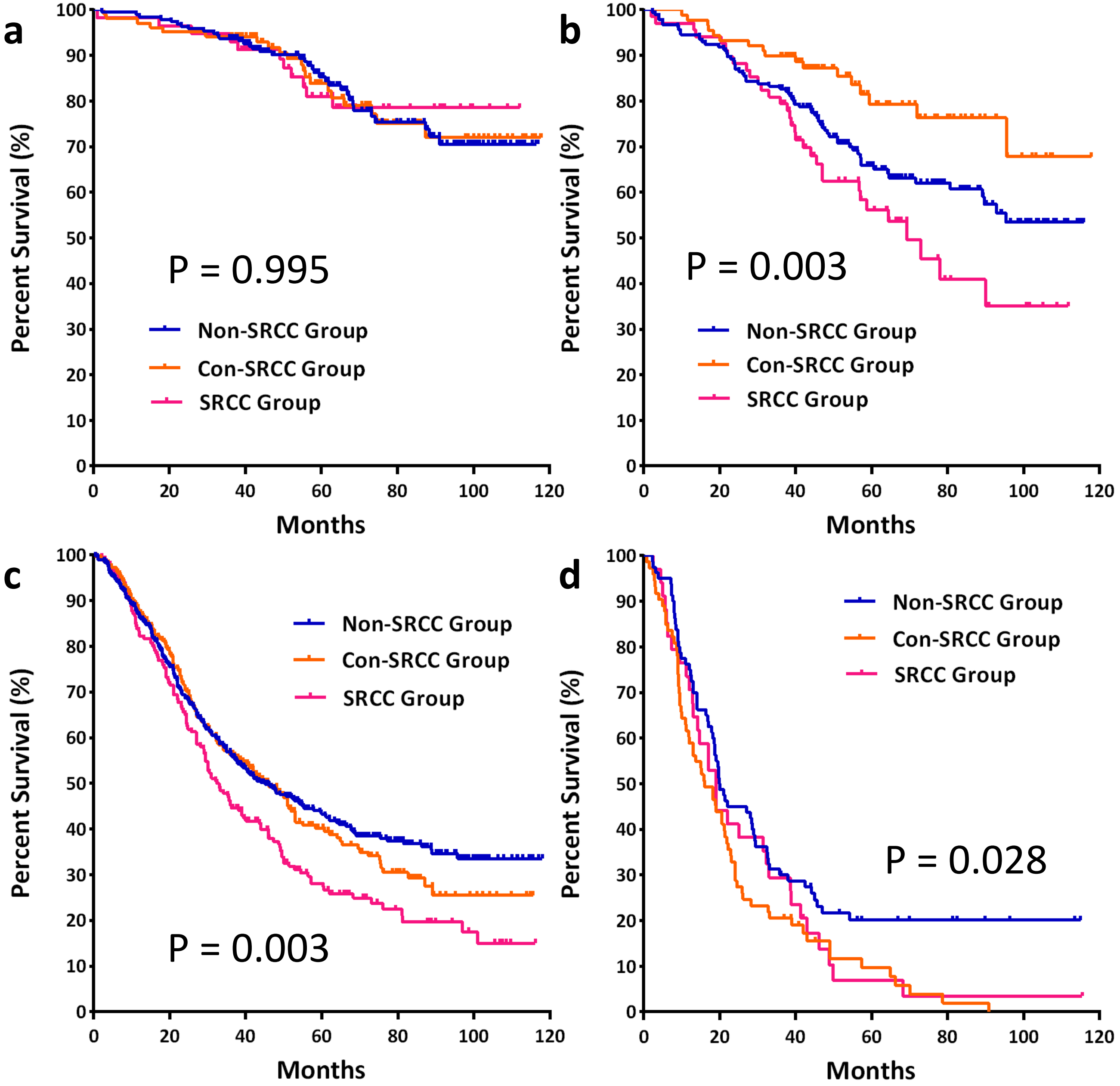

Supplementary Figure 2: Survival outcomes in gastric cancer patients with different signet-ring cell frequency Survival curves of patients among the non-SRCC, con-SRCC, and SRCC in Stage I (**a**), stage II (**b**), stage III (**c**), Stage IV (**d**). *P* value was estimated by using Cox Model.

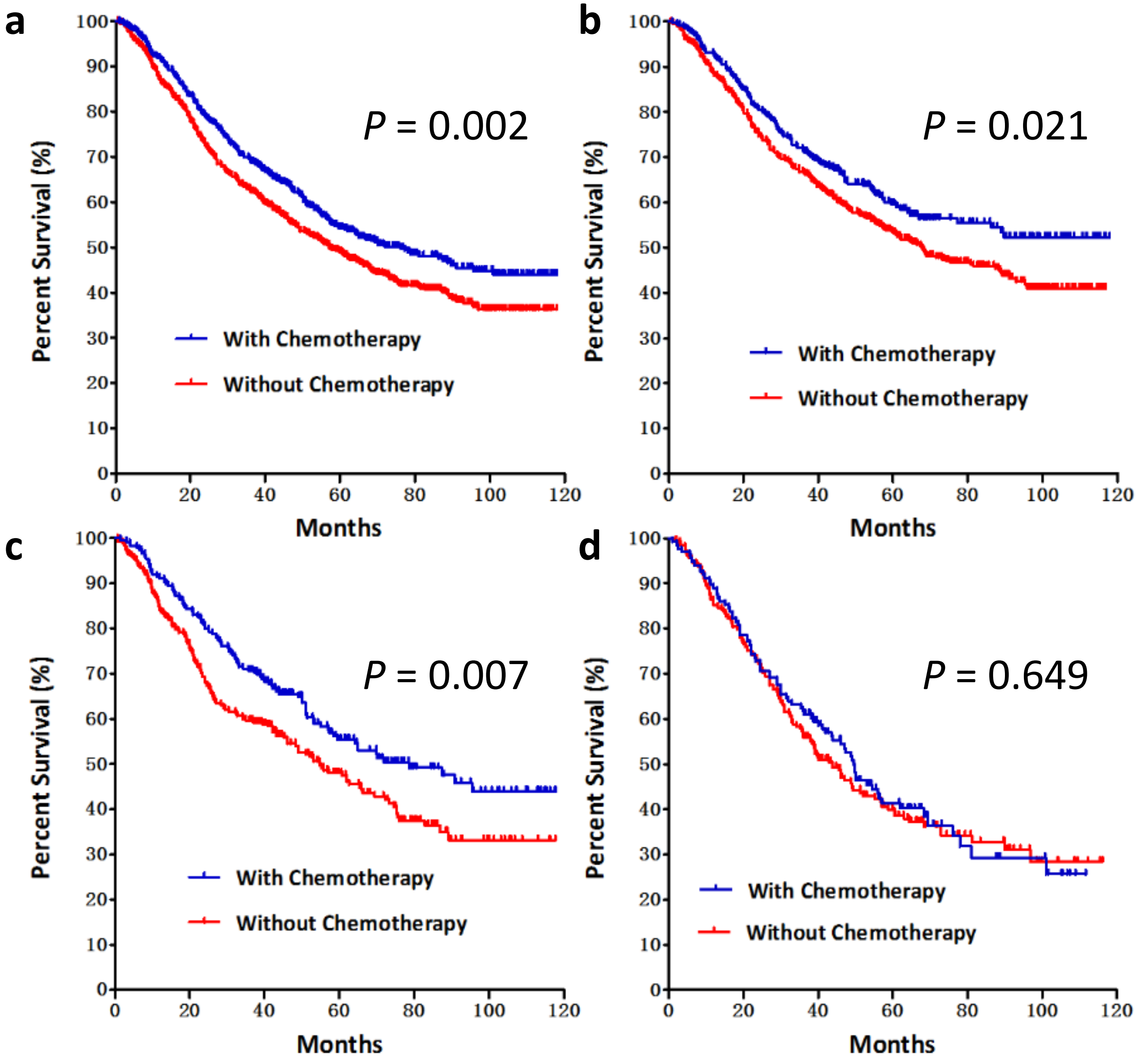

Supplementary Figure 3: Relationship between survival outcomes and chemotherapy treatment. Survival curves with/without chemotherapy treatment in all patients (**a**), non-SRCC (**b**), con-SRCC (**c**), and SRCC (**d**). *P* value was estimated by using Cox Model.

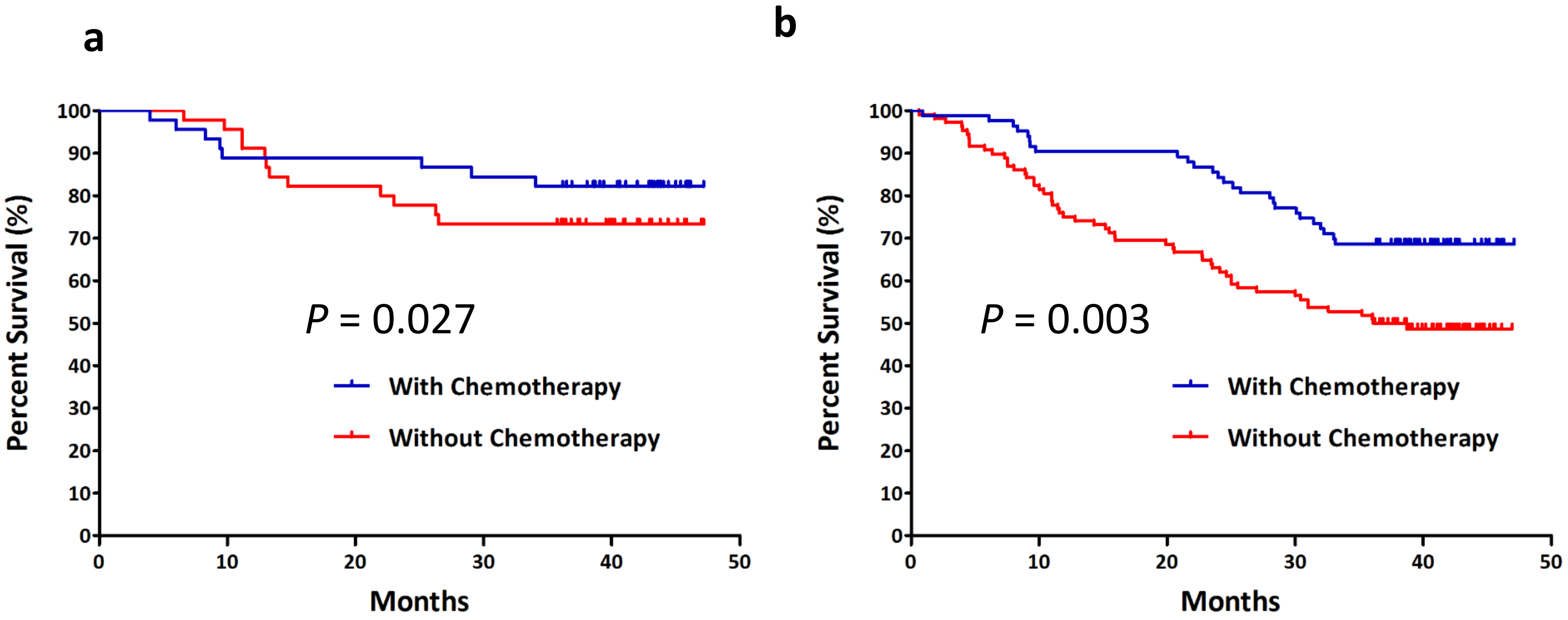

Supplementary Figure 4: Relationship between survival outcomes and chemotherapy treatment. Survival curves with/without chemotherapy treatment in Intestinal type (N = 90) (**a**) and Diffuse type (N = 191) (**b**) are illustrated in patients with Lauren's subtypes since 2012, when such classification has been routinely evaluated in West China Hospital. *P* value was estimated by using Cox model adjusted for TNM stage.

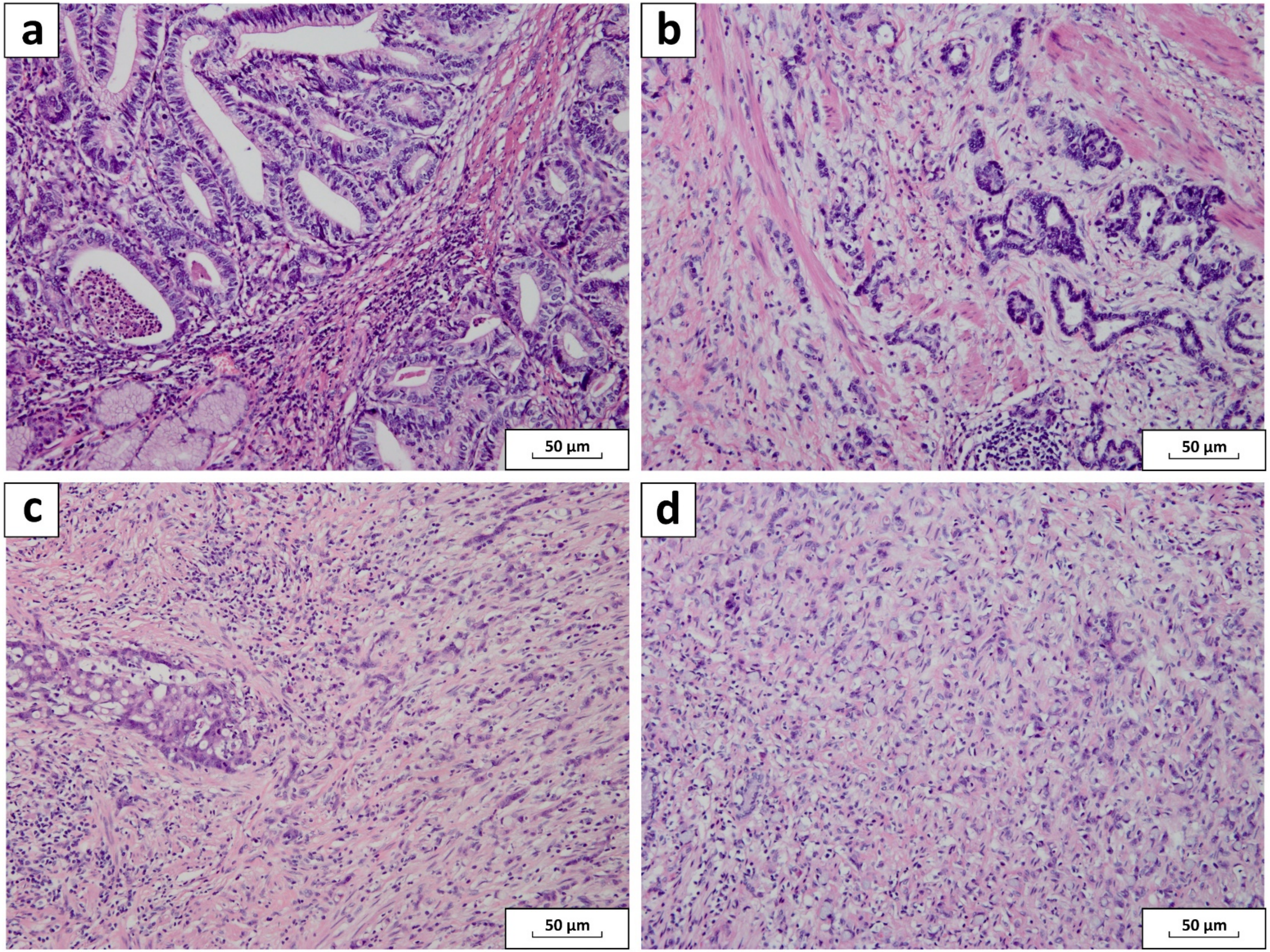

Supplementary Figure 5: Percentage of signet-ring cells among 4 groups. Microscopic characteristics of tumor samples with hematoxylin/eosin staining in (**a**) non-SRCC (with no signet-ring cell), (**b**) con-SRCC (with < 50% presence of signet-ring cells), (**c**) SRCC (with > 50% presence of signet-ring cells), and (**d**) HSRCC (with > 80% presence of signet-ring cells).

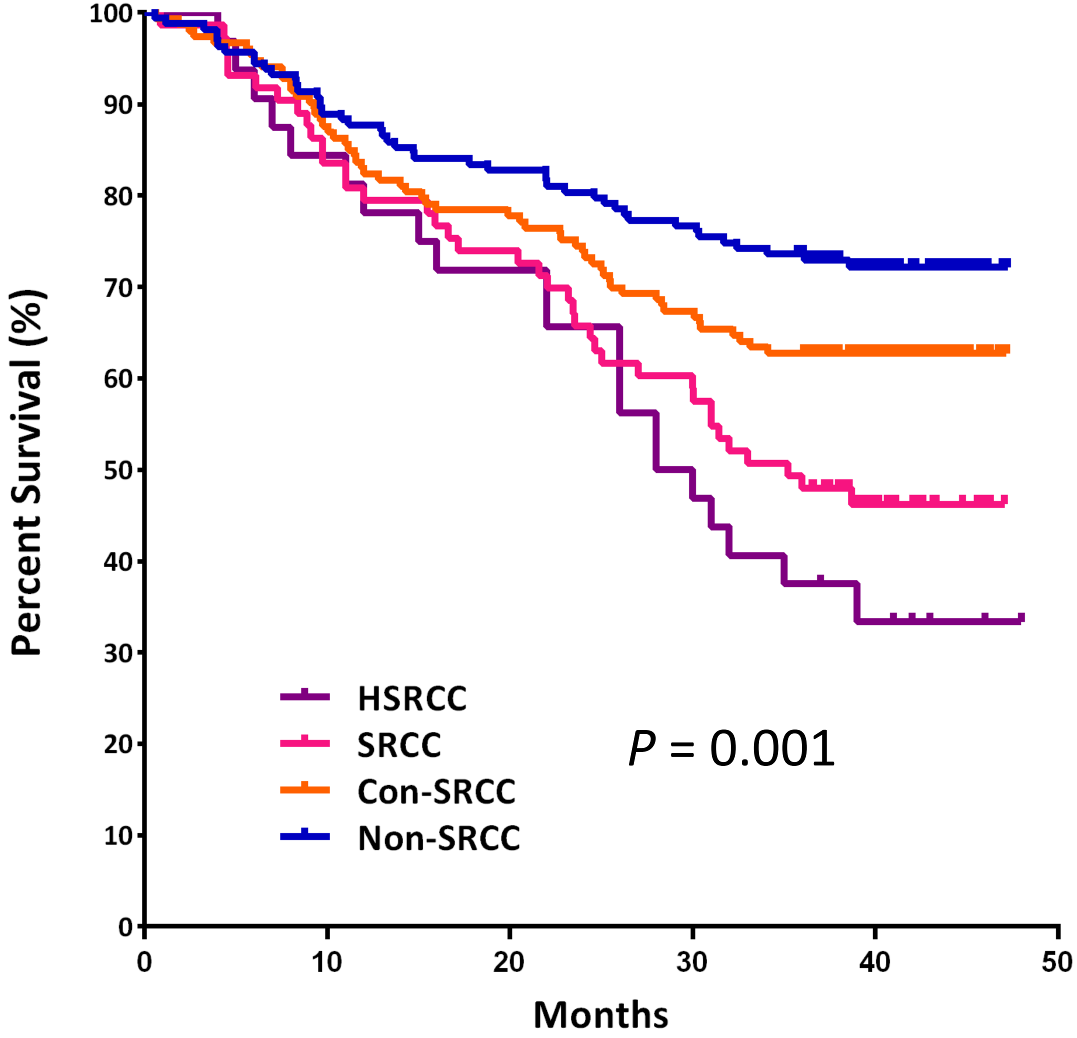

Supplementary Figure 6: Survival outcomes in gastric cancer patients with different signet-ring cell frequency (2012). Survival curves of the patients in 2012 among the four groups the non-SRCC, con-SRCC, SRCC and HSRCC. *P* value was estimated among non-SRCC, con-SRCC, and SRCC only by using Cox Model.

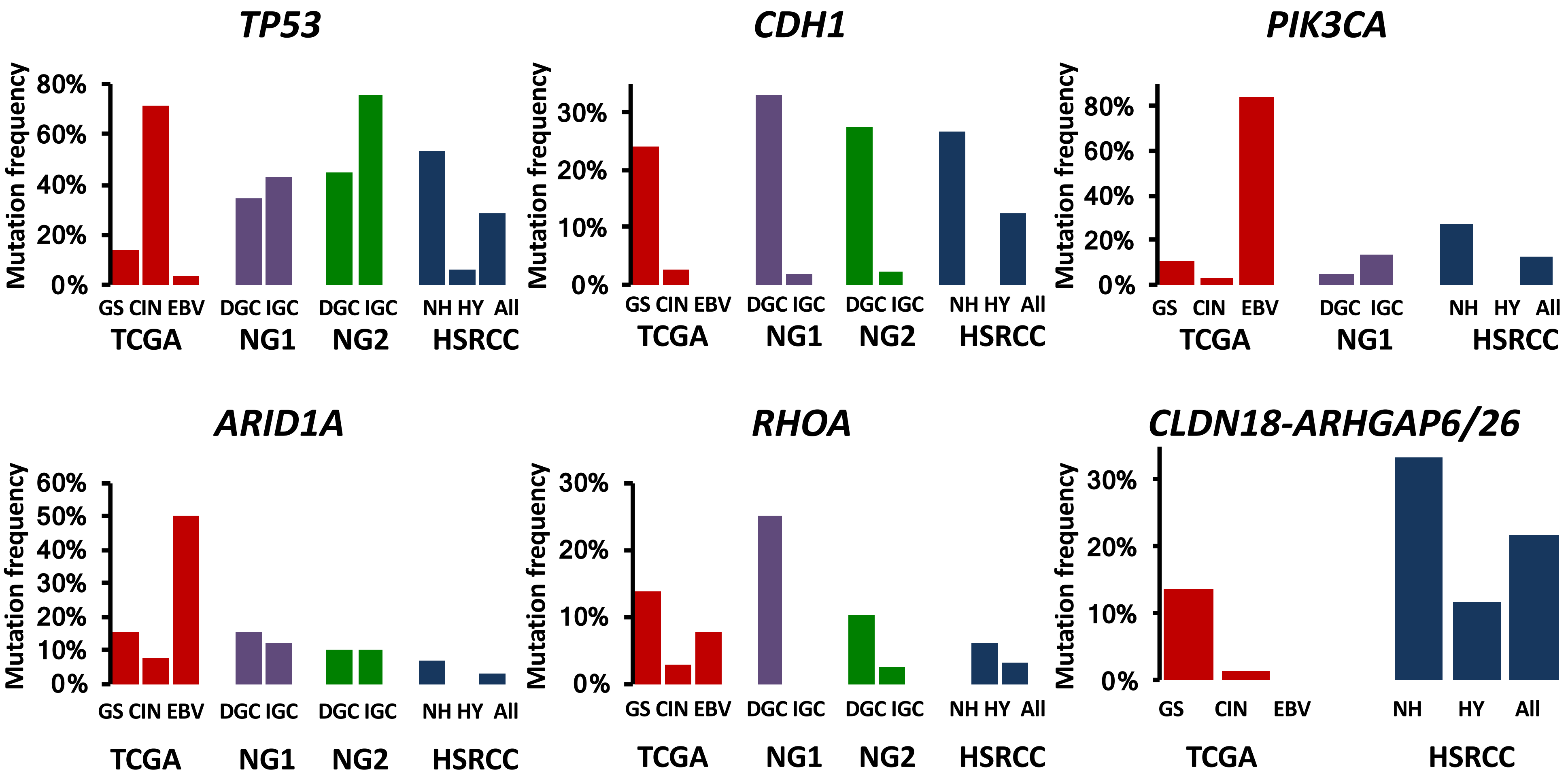

Supplementary Figure 7: Frequency of the common gene alterations in different studies. Mutation rate were retrieved from 4 studies, including TCGA (ref3, N = 58, 147, and 26 for GS, CIN and EBV subtypes, respectively), NG1 (ref7, N = 87 and 51 for DGC and IGC, respectively), NG2 (ref11, N = 29 and 41 for DGC and IGC, respectively), and our HSRCC study (N = 15, 17 and 32 for NH, HY, and All, respectively). GS = genomically stable; CIN = chrom instability; EBV = EBV-CIMP; DGC = Diffuse type of gastric cancer; IGC = intestinal type of gastric cancer; NH = non-hypomutant; HY = hypomutant.

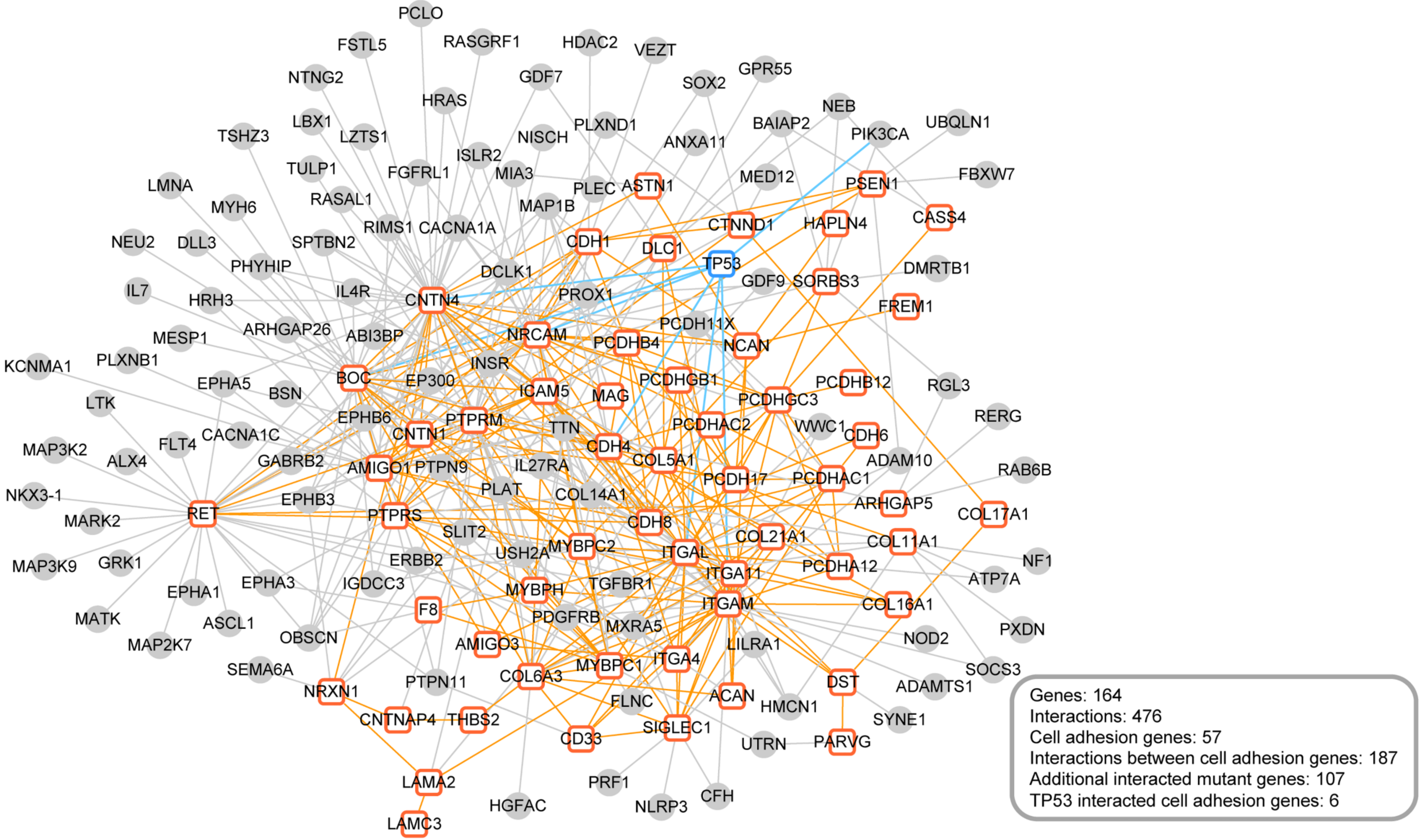

Supplementary Figure 8: The PPI network of mutant cell adhesion genes. 57 cell adhesion genes (orange) formed 187 interactions. 107 additional mutant genes (gray) also have interaction with these cell adhesion genes. TP53 (blue) is also involved in the cell adhesion subnetwork.

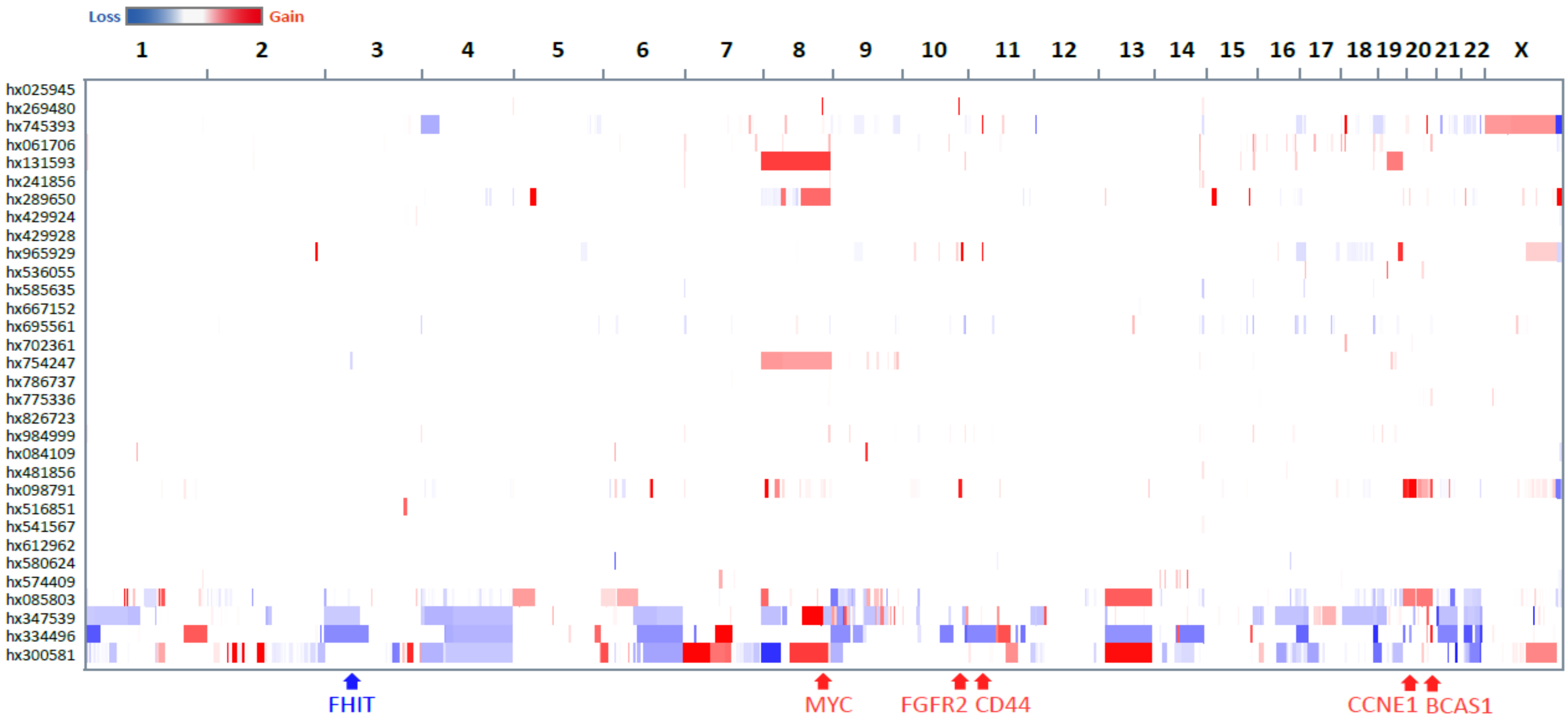

Supplementary Figure 9: somatic copy number alterations of HSRCC patients: Overall SCNAs were illustrated for each patients listed on y-axis, and all chromosomes (except Y) on the x-axis. Loss and gain were shown in blue and red, respectively.

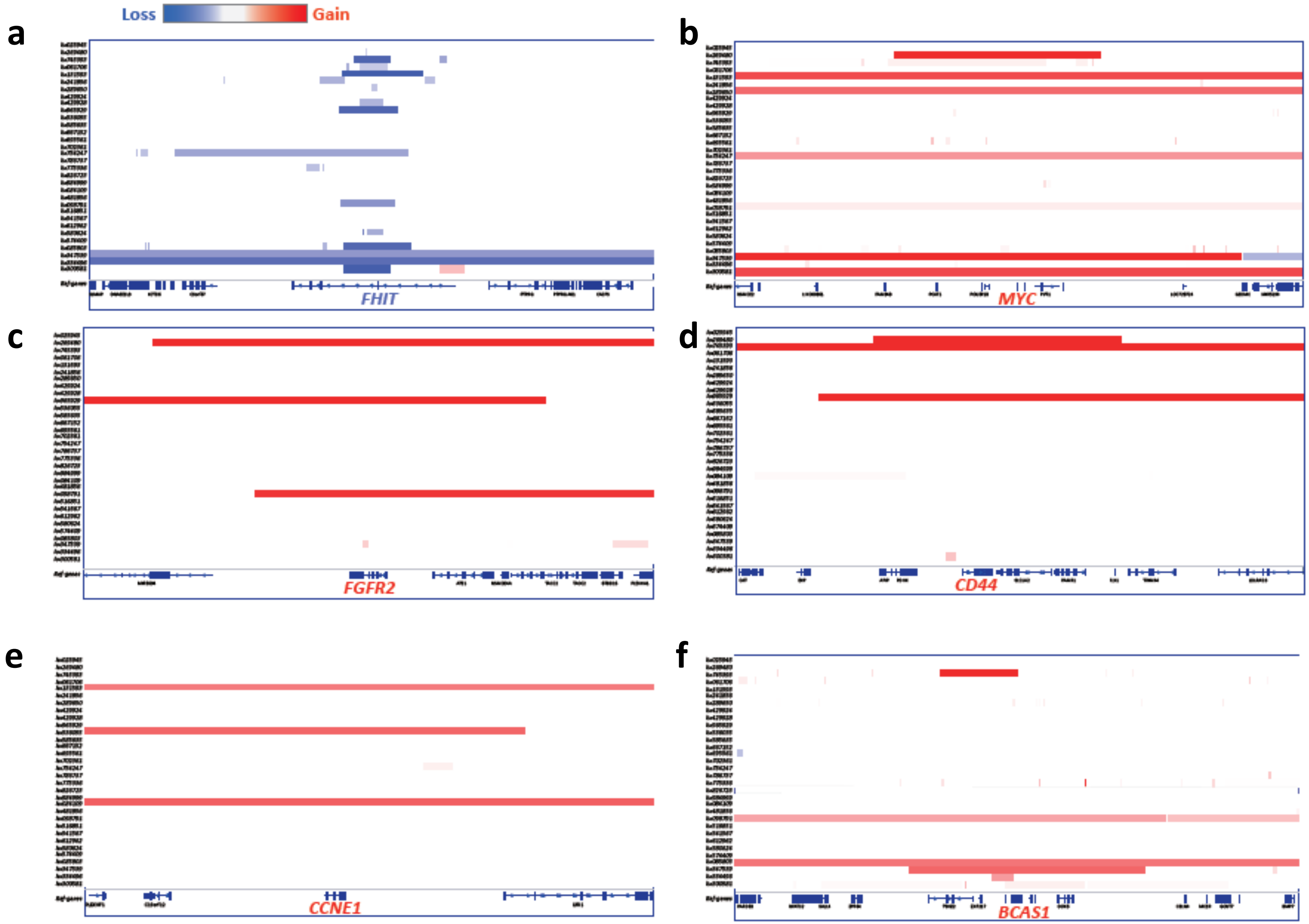

Supplementary Figure 10: Recurrent somatic copy number variations in HSRCC patients. foci deletion of (**a**) FHIT, overlapped regions of gain at (**b**) 8q24.21 (including *MYC*), (**c**) 10q26.13 (including *FGFR2*), (**d**) 11p13 (including *CD44*), (**e**) 19q12 (including *CCNE1*), and (**f**) 20q13.2 (including *BCAS1*).

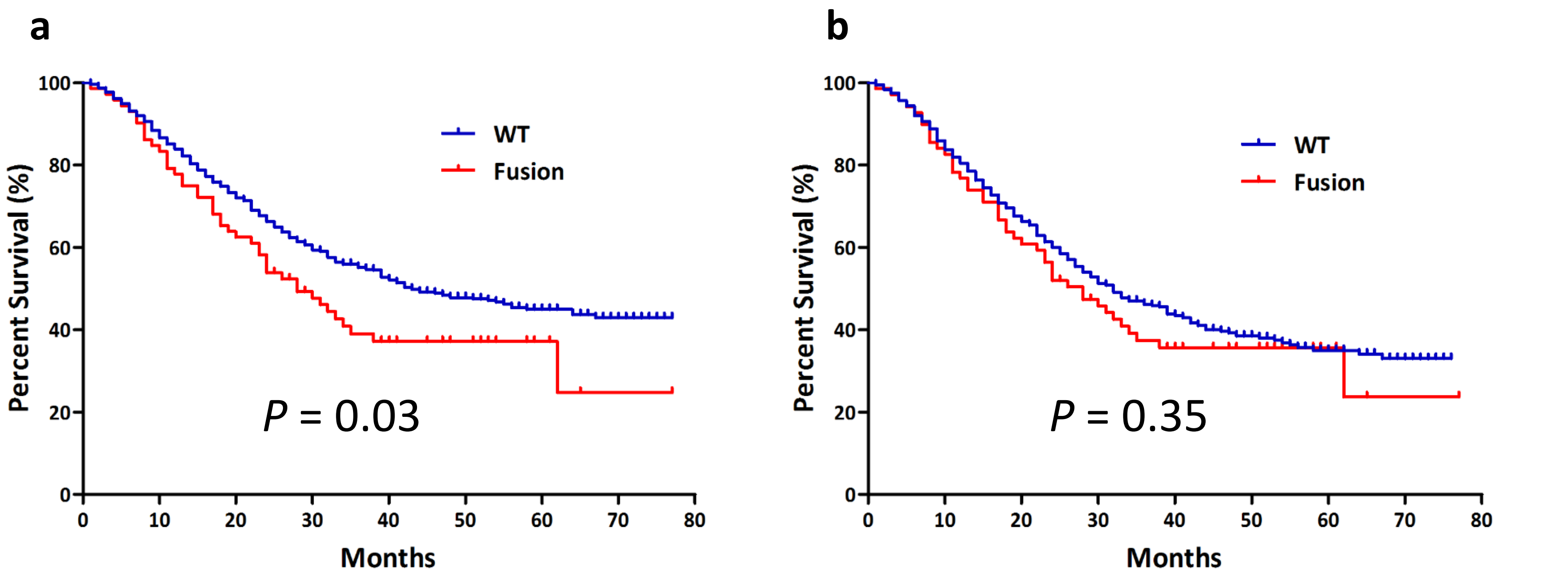

Supplementary Figure 11: Survival outcomes in all gastric cancer patients (**a**) and Stage III/IV gastric cancer patients (**b**) with *CLDN18-ARHGAP26/6* fusion. Survival curves of patients with *CLDN18-ARHGAP26/6* and fusion-free. *P* value was estimated by using Cox Model; WT: wildtype

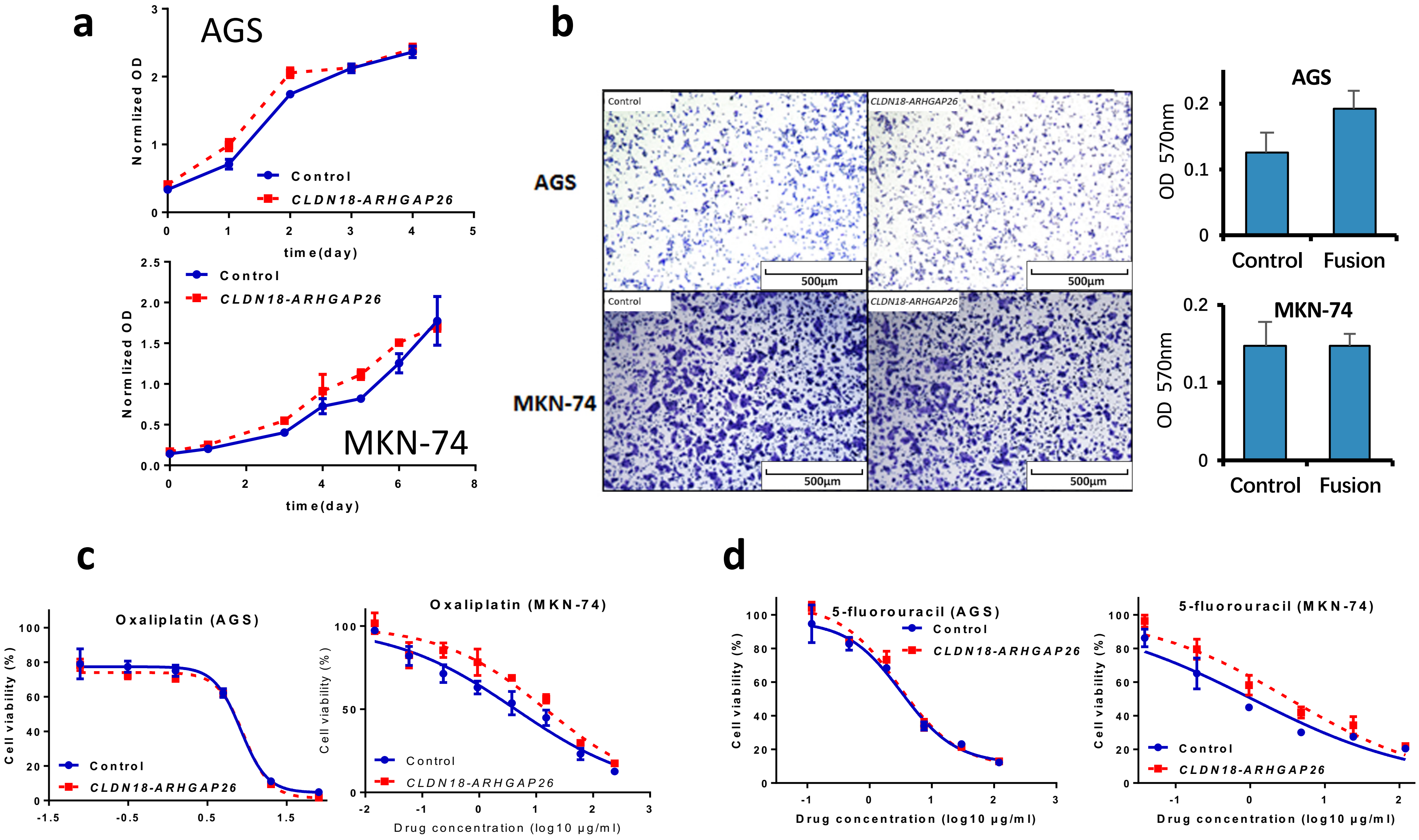

Supplementary Figure 12: Impact of *CLDN18-ARHGAP26* overexpression in AGS and MKN-74. Cell proliferation (**a**), cell migration (**b**), and drug response to oxaliplatin (**c**) 5-fluorouracil (**d**) was estimated in *CLDN18-ARHGAP26* stably expressed AGS/MKN-74 cells and their matched control. AGS and MKN-74 were treated for 2 and 3 days, respectively. Control: empty control. Each experiment has been replicated for three times, and data is presented as mean ± SD

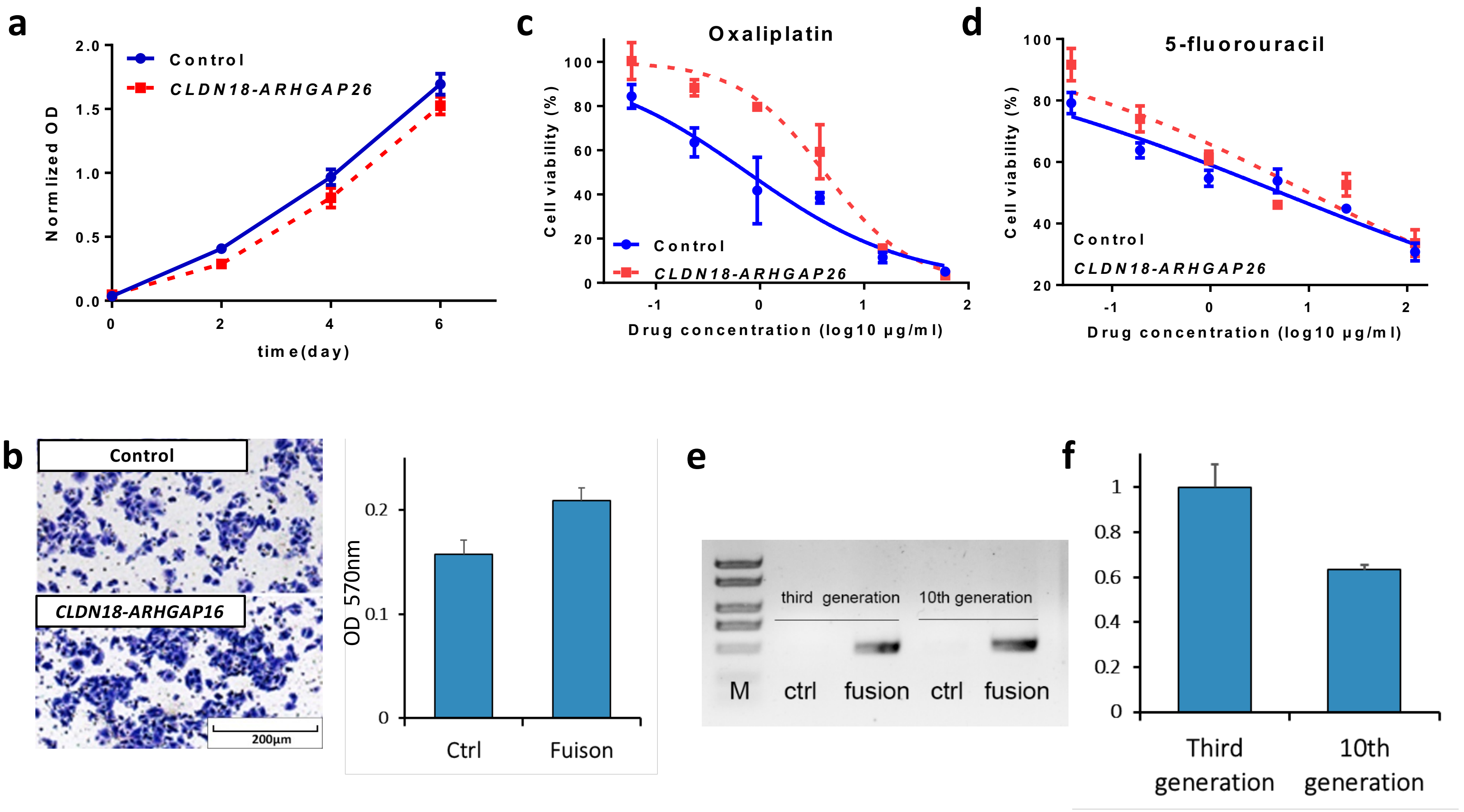

Supplementary Figure 13: Introduction of *CLDN18-ARHGAP26* in cell lines. Cell proliferation (**a**), cell migration (**b**), and drug response to oxaliplatin (**c**) 5-fluorouracil (**d**) was estimated in *CLDN18-ARHGAP26* stably expressed BGC-823 cells and their matched control with retrovirus infection system. Expression level of *CLDN18-ARHGAP26* in AGS cells was estimated by reverse-transcript PCR (**e**) or qPCR (**f**) for different generations. Each experiment has been replicated for three times, and data is presented as mean ± SD; Ctrl: empty control.

Supplementary Table 1: Characteristics of all patients (2006-2012)

Variables	Characteristics	All patients N = 1868 (%)
**SRC Status**	**Non-SRCC**	937 (50.2)
	**Con-SRCC**	556 (29.8)
	**SRCC**	375 (20.0)
**Age**	**Years**	57.2±11.8
**Gender**	**Male**	1307 (70.0)
	**Female**	561 (30.0)
**Tumor Size**	**cm**	5.4±3.1
**Tumor Location**	**U**	504 (27.0)
	**M**	213 (11.4)
	**L**	1106 (59.2)
	**UML**	45 (2.4)
**Tumor Grade**	**G1-2**	367 (19.6)
	**G3-4**	1501 (80.4)
**Residual Degree**	**R0**	1643 (88.0)
	**R1/R2**	225 (12.0)
**T stage**	**T1**	343 (18.4)
	**T2**	232 (12.4)
	**T3**	141 (7.5)
	**T4**	1152 (61.7)
**N stage**	**N0**	554 (29.7)
	**N1**	341 (18.3)
	**N2**	305 (16.3)
	**N3**	668 (35.8)
**M stage**	**M0**	1664 (89.1)
	**M1**	204 (10.9)
**TNM stage**	**I**	396 (21.2)
	**II**	368 (19.7)
	**III**	900 (48.2)
	**IV**	204 (10.9)
**Chemotherapy**	**with**	721 (38.6)
	**Without**	1147 (61.4)
**Lauren's Classification**	**Intestinal**	94 (5.0)
	**Diffuse**	197 (10.6)
	**Unknown**	1577 (84.4)
**Nervous invasion†**	**Negative**	1723 (92.2)
	**Positive**	145 (7.8)
**Capillary invasion†**	**Negative**	1487 (79.6)
	**Positive**	381 (20.4)
**Extranodal metastasis†**	**Negative**	1625 (87.0)
	**Positive**	243 (13.0)

†According the Japanese classification of gastric carcinoma: 3rd English edition.

Abbreviations: non-SRCC, cancers without signet-ring cells; con-SRCC, cancers with <50% presence of signet-ring cells; SRCC, cancers with >50% presence of signet-ring cells; U, upper; M, middle, L, lower.

Supplementary Table 2: Comparison of the Clinicopathological characteristics of patients of non-SRCC, con-SRCC, and SRCC (2006-2012)

Variables	Characteristics	Non-SRCC N = 937 (%)	Con-SRCC N = 556 (%)	SRCC N = 375 (%)	*P* value
Age	Years	59.6 ± 10.7	55.3 ± 12.1	54.0 ± 12.7	< 0.001
Gender	Male	725 (77.4)	373 (67.1)	209 (55.7)	< 0.001
	Female	212 (22.6)	183 (32.9)	166 (44.3)	
Tumor Size	cm	5.2 ± 2.7	5.5 ± 3.3	5.7 ± 3.6	0.242
Longitudinal Location	U	321 (34.3)	122 (21.9)	61 (16.3)	< 0.001
	M	113 (12.1)	56 (10.1)	44 (11.7)	
	L	494 (52.7)	362 (65.1)	250 (66.7)	
	UML	9 (1.0)	16 (2.9)	20 (5.3)	
Tumor Grade	G1-2	351 (37.5)	16 (2.9)	0 (0)	< 0.001
	G3-4	586 (62.5)	540 (97.1)	375 (100)	
Residual Degree	R0	838 (89.4)	482 (86.7)	323 (86.1)	0.139
	R1/R2	99 (10.6)	74 (13.3)	52 (13.9)	
pT stage	T1	183 (19.5)	99 (17.8)	61 (16.3)	0.003
	T2	132 (14.1)	69 (12.4)	31 (8.3)	
	T3	77 (8.2)	40 (7.2)	24 (6.4)	
	T4	545 (58.2)	348 (62.6)	259 (69.1)	
pN stage	N0	320 (34.2)	141 (25.4)	93 (24.8)	< 0.001
	N1	198 (21.1)	83 (14.9)	60 (16.0)	
	N2	171 (18.2)	81 (14.6)	53 (14.1)	
	N3	248 (26.5)	251 (45.1)	169 (45.1)	
pM stage	M0	847 (90.4)	480 (86.3)	337 (89.9)	0.045
	M1	90 (9.6)	76 (13.7)	38 (10.1)	
pTNM stage	I	224 (23.9)	112 (20.1)	60 (16.0)	< 0.001
	II	203 (21.7)	92 (16.5)	73 (19.5)	
	III	420 (44.8)	276 (49.6)	204 (54.4)	
	IV	90 (9.6)	76 (13.7)	38 (10.1)	
Nervous invasion†	Negative	891 (95.1)	502 (90.3)	330 (88.0)	< 0.001
	Positive	46 (4.9)	54 (9.7)	45 (12.0)	
Capillary invasion†	Negative	756 (80.7)	432 (77.7)	299 (79.7)	0.383
	Positive	181 (19.3)	124 (22.3)	76 (20.3)	
Extranodal metastasis†	Negative	826 (88.2)	485 (87.2)	314 (83.7)	0.097
	Positive	111 (11.8)	71 (12.8)	61 (16.3)	

†According the Japanese classification of gastric carcinoma: 3rd English edition.

Abbreviations:non-SRCC, cancers without signet-ring cells; con-SRCC, cancers with <50% presence of signet-ring cells; SRCC, cancers with >50% presence of signet-ring cells; U, upper; M, middle, L, lower. *P* value was calculated by the comparison among the Non-SRCC, Con-SRCC and SRCC groups, age and tumor size were tested by one-way ANOVA test, tumor grade and tumor stage were tested by Kruskal-Wallis test and others were tested by Pearson's Chi-square test.

Supplementary Table 3: Clinicopathological characteristics of patients of non-SRCC, con-SRCC, SRCC and HSRCC patients (2012)

Variables	Characteristics	Non-SRCC N = 168 (%)	Con-SRCC N = 156 (%)	SRCC N = 77 (%)	HSRCC N = 32 (%)	*P* value*
**Age**	**Years**	61.4±9.9	56.3±12.2	55.3±11.7	52.9±13.3	< 0.001
**Gender**	**Male**	130 (77.4)	108 (69.2)	44 (57.1)	25 (78.1)	0.005
	**Female**	38 (22.6)	48 (30.8)	33 (42.9)	7 (21.9)	
**Tumor Size**	**cm**	5.2±2.8	5.9±2.9	5.9±2.8	7.4±2.8	0.061
**Tumor Location**	**U**	71 (42.3)	41 (26.3)	19 (24.7)	4 (12.5)	< 0.001
	**M**	26 (15.5)	8 (5.1)	8 (10.4)	6 (18.8)	
	**L**	70 (41.7)	104 (66.7)	48 (62.3)	19 (59.4)	
	**UML**	1 (0.6)	3 (1.9)	2 (2.6)	3 (9.4)	
**Tumor Grade**	**G1-2**	72 (42.9)	2 (1.3)	0 (0)	0 (0)	< 0.001
	**G3-4**	96 (57.1)	154 (98.7)	77 (100)	32 (100)	
**Residual Degree**	**R0**	149 (88.7)	134 (85.9)	66 (85.7)	28 (87.5)	0.703
	**R1/R2**	19 (11.3)	22 (14.1)	11 (14.3)	4 (12.5)	
**pT stage**	**T1**	41 (24.4)	19 (12.2)	9 (11.7)	1 (3.1)	0.001
	**T2**	25 (14.9)	19 (12.2)	9 (11.7)	1 (3.1)	
	**T3**	26 (15.5)	24 (15.4)	7 (9.1)	2 (6.2)	
	**T4**	76 (45.2)	94 (60.3)	52 (67.5)	28 (87.5)	
**pN stage**	**N0**	73 (43.5)	34 (21.8)	12 (15.6)	5 (15.6)	< 0.001
	**N1**	35 (20.8)	18 (11.5)	15 (19.5)	1 (3.1)	
	**N2**	29 (17.3)	19 (12.2)	13 (16.9)	2 (6.2)	
	**N3**	31 (18.5)	85 (54.5)	37 (48.1)	24 (75.0)	
**pM stage**	**M0**	160 (95.2)	135 (86.5)	71 (92.2)	26 (81.2)	0.021
	**M1**	8 (4.8)	21 (13.5)	6 (7.8)	6 (18.8)	
**pTNM stage**	**I**	52 (31.0)	22 (14.1)	8 (10.4)	2 (6.2)	< 0.001
	**II**	38 (22.6)	28 (17.9)	18 (23.4)	2 (6.2)	
	**III**	70 (41.7)	85 (54.5)	45 (58.4)	22 (68.8)	
	**IV**	8 (4.8)	21 (13.5)	6 (7.8)	6 (18.8)	
**Nervous invasion†**	**Negative**	158 (94.0)	135 (86.5)	73 (94.8)	29 (90.6)	0.027
	**Positive**	10 (6.0)	21 (13.5)	4 (5.2)	3 (9.4)	
**Capillary invasion†**	**Negative**	151 (89.9)	124 (79.5)	66 (85.7)	30 (93.8)	0.032
	**Positive**	17 (10.1)	32 (20.5)	11 (14.3)	2 (6.2)	
**Extranodal metastasis†**	**Negative**	158 (94.0)	141 (90.4)	67 (87.0)	27 (84.4)	0.171
	**Positive**	10 (6.0)	15 (9.6)	10 (13.0)	5 (15.6)	
**Lauren Type**	**Intestinal**	83 (49.4)	11 (7.1)	0 (0)	0 (0)	< 0.001
	**Diffuse**	15 (8.9)	117 (75.0)	65 (84.4)	32 (100)	
	**Unknown**	70 (41.7)	28 (17.9)	12 (15.6)	0 (0)	
**Chemotherapy**	**With**	72 (42.9)	73 (46.8)	30 (39.0)	17 (53.1)	0.507
	**Without**	96 (57.1)	83 (53.2)	47 (61.0)	15 (46.9)	

†According the Japanese classification of gastric carcinoma: 3rd English edition.

Abbreviations: :non-SRCC, cancers without signet-ring cells; con-SRCC, cancers with <50% presence of signet-ring cells; SRCC, cancers with >50% presence of signet-ring cells; U, upper; M, middle, L, lower.

**P* value was calculated by the comparison among the Non-SRCC, Con-SRCC and SRCC groups, age and tumor size were tested by one-way ANOVA test, tumor grade and tumor stage were tested by Kruskal-Wallis test and others were tested by Pearson's Chi-square test.

Supplementary Table 4: Correlation of clinical characteristics with the presence of *CLDN18-ARHGAP26/6* fusions in patients

Variables	West China (N = 829)				TCGA (N = 295)	
	Univariate		Multivariate†			
	OR (95% CI)	*P* value	OR (95% CI)	*P* value	OR (95% CI)	*P* value
Gender (female *vs.* male)	4.67 (2.83-7.72)	1.7×10^{-9}	2.81 (1.51-5.24)	7.8×10^{-6}	3.67(1.10-12.22)	0.034
Age (years)	0.94 (0.92-0.96)	4.2×10^{-10}	0.95 (0.92-0.97)	0.0001	0.99(0.98-1.00)	0.034
Lauren's Classification	1.79 (1.19-2.69)	0.005	1.59 (0.82-3.06)	NA	0.08(0.02-0.38)	0.001
SRCC status	2.62 (1.90-3.62)	4.1×10^{-9}	1.82 (1.12-2.97)	0.0007	NA	NA
T stage	1.35 (0.92-1.98)	0.13	0.91 (0.48-1.71)	0.19	0.96(0.46-1.96)	0.9
N stage	3.60 (2.22-5.84)	2.2×10^{-7}	4.17 (1.91-9.07)	1.4×10^{-5}	1.42(0.88-2.31)	0.15
M stage	2.34 (1.34-4.07)	0.003	7.08 (1.10-45.51)	0.43	1.10(0.14-8.89)	0.93
TNM stage	2.40 (1.61-3.57)	1.7×10^{-5}	0.19 (0.03-1.07)	NA	1.14(0.84-1.56)	0.4

Enrichment of patients with *CLDN18-ARHGAP26/6* fusions in terms of clinical characteristicswere estimated in both TCGA and our patients, † all clinical characteristics have been adjusted in the multivariate analyese except Lauren's classification and TNM stage (due to multicollinearity). P value and ORs were estimated by the logistic regression model. Abbreviations: OR, Odds ratio; 95% CI, 95% confidence interval of the risk ratio

Supplementary Table 5: Statistics of whole genome sequencing data from 32 HSRCC patients

Samples	Normal Control					Tumor				
	**Total Reads Number(M)**	**Reads Mapping Rate**	**Mean Depth**	**Reference Genome coverag**	**PCR Duplication Rate**	**Total Reads Number(M)**	**Reads Mapping Rate**	**Mean Depth**	**Reference Genome coverag**	**PCR Duplication Rate**
hx745393	849.32	0.9932	42.9	0.9983	0.1018	1388.44	0.9968	70.5	0.9986	0.1091
hx061706	959.37	0.9974	48.7	0.9985	0.1033	1408.22	0.9979	71.8	0.9987	0.2147
hx131593	856.4	0.9956	43.5	0.9918	0.1055	1456.42	0.9979	74.3	0.9924	0.2136
hx241856	1019.26	0.9961	51.8	0.9919	0.117	1748.81	0.998	89.3	0.9925	0.2276
hx289650	844.25	0.9963	42.9	0.9981	0.1099	1344.25	0.9972	68.2	0.9984	0.1373
hx429924	722.36	0.996	36.6	0.998	0.1082	1354.66	0.9973	68.8	0.9984	0.1341
hx429928	663.09	0.9931	33.5	0.9979	0.1051	1173.86	0.997	59.6	0.9983	0.1248
hx965929	879.77	0.9949	44.5	0.9981	0.1247	1316.26	0.9975	66.9	0.9984	0.1322
hx536055	798.29	0.9921	40.3	0.9979	0.1103	1228.96	0.9967	62.4	0.9982	0.1233
hx585635	785.74	0.9958	39.9	0.9918	0.2175	1205.54	0.9977	61.4	0.9922	0.1239
hx667152	787.23	0.9951	39.9	0.9981	0.2128	1157.05	0.9969	58.8	0.9984	0.1204
hx695561	745.15	0.9944	37.8	0.9981	0.2156	1192.95	0.9975	60.7	0.9984	0.1256
hx702361	655.24	0.9884	32.8	0.998	0.0828	1184.04	0.9961	60	0.9984	0.1248
hx754247	713.31	0.9963	36.2	0.9916	0.0692	1204.18	0.9979	61.4	0.9921	0.1175
hx786737	813.61	0.9929	41.1	0.9978	0.0841	1267.95	0.9969	64.5	0.9981	0.1246
hx775336	736.97	0.9951	37.3	0.9977	0.0758	1184.04	0.9974	60.2	0.9981	0.1144
hx826723	779.23	0.9946	39.4	0.9981	0.0903	1493.84	0.9977	76.1	0.9985	0.1421
hx984999	774.48	0.9951	39.2	0.992	0.0796	1254.44	0.9978	64	0.9925	0.121
hx084109	757.72	0.9952	38.4	0.9982	0.0788	1264.25	0.9975	64.3	0.9985	0.1201
hx481856	789.18	0.9938	39.9	0.9916	0.0875	1400.69	0.9972	71.2	0.9921	0.1464
hx098791	691.01	0.9896	34.7	0.998	0.0929	1358.01	0.9968	68.9	0.9985	0.1469
hx085803	605.8	0.9944	30.6	0.9979	0.1294	1293.77	0.9971	65.7	0.9985	0.1478
hx300581	737.98	0.9971	37.4	0.998	0.1301	1241.47	0.9975	63.2	0.9983	0.1398
hx334496	880.94	0.9978	44.7	0.9981	0.1405	1325.77	0.9982	67.5	0.9984	0.1473
hx347539	741.54	0.997	37.6	0.9981	0.1324	1221.62	0.9973	62.1	0.9985	0.1358
hx516851	809.85	0.9978	41.1	0.9982	0.1363	1234.35	0.9978	62.7	0.9985	0.1439
hx541567	731.78	0.9977	37.1	0.9981	0.1326	1127.05	0.9977	57.2	0.9985	0.1431
hx612962	872.41	0.9978	44.4	0.992	0.1399	1359.45	0.998	69.2	0.9923	0.1528
hx574409	795.15	0.9979	40.4	0.9981	0.1348	1148.31	0.9979	58.4	0.9983	0.145
hx580624	807.67	0.9956	41	0.9919	0.1367	1222.61	0.9981	62.3	0.9923	0.1462
hx025945	897.33	0.9939	45.4	0.9983	0.1024	1360.41	0.9966	69.1	0.9985	0.1097
hx269480	1027.09	0.9961	52.1	0.9982	0.1143	1636.64	0.9978	83.3	0.9984	0.1207
