# Supplementary material for: Prognostic significance of frequent CLDN18-ARHGAP26/6 fusion in gastric signet-ring cell cancer
Source: Nat Commun. 2018 Jun 30;9:2447. doi: 10.1038/s41467-018-04907-0 (PMC6026495; doi:10.1038/s41467-018-04907-0)
Supplement: Supplementary file 3 — Description of Additional Supplementary Files [file 41467_2018_4907_MOESM3_ESM.pdf]

Description of Additional Supplementary Files:

Supplementary data 1: all functional SNVs and indels identified by WGS

Supplementary data 2: Characteristics of 32 HSRCC patients

Supplementary data 3: DAVID prediction of all altered genes (SNV/INDEL only) in non-hypo mutation group

Supplementary data 4: DAVID prediction of all altered genes in hypo mutation group

Supplementary data 5: SVs identified by whole-genome sequencing

Supplementary data 6: Characteristics of 797 validation patients

Supplementary data 7: DAVID prediction of all altered genes (SNV/INDEL, SVs, and SCNA) in non-hypo mutation group
